# Supplementary material for: Deep learning guided design of protease substrates
Source: Nat Commun. 2026 Jan 6;17:54. doi: 10.1038/s41467-025-67226-1 (PMC12775482; doi:10.1038/s41467-025-67226-1)
Supplement: Supplementary file 1 — Supplementary Information [file 41467_2025_67226_MOESM1_ESM.pdf]

Supplementary Information for:

Deep learning guided design of protease substrates

Carmen Martin-Alonso[1,2,†], Sarah Alamdari[3,†], Tahoura S. Samad[1], Kevin K. Yang[3], Sangeeta N. Bhatia[1,2,4-9,*], Ava P. Amini[3,*]

[1]Koch Institute for Integrative Cancer Research, MIT, Cambridge, MA, USA

[2]Harvard-MIT Division of Health Sciences and Technology, Cambridge, MA, USA

[3]Microsoft Research, Cambridge, MA, USA

[4]Institute for Medical Engineering and Science, MIT, Cambridge, MA, USA

[5]Marble Center of Cancer Nanomedicine, MIT, Cambridge, MA, USA

[6]Broad Institute of MIT and Harvard, Cambridge, MA, USA

[7]Electrical Engineering and Computer Science, MIT, Cambridge, MA, USA

[8]Wyss Institute at Harvard University, Boston, MA, USA

[9]Howard Hughes Medical Institute, Cambridge, MA, USA

[†]These authors contributed equally to this work

[*]These authors jointly supervised the work

[*]Correspondence to: sbhatia@mit.edu, ava.amini@microsoft.com

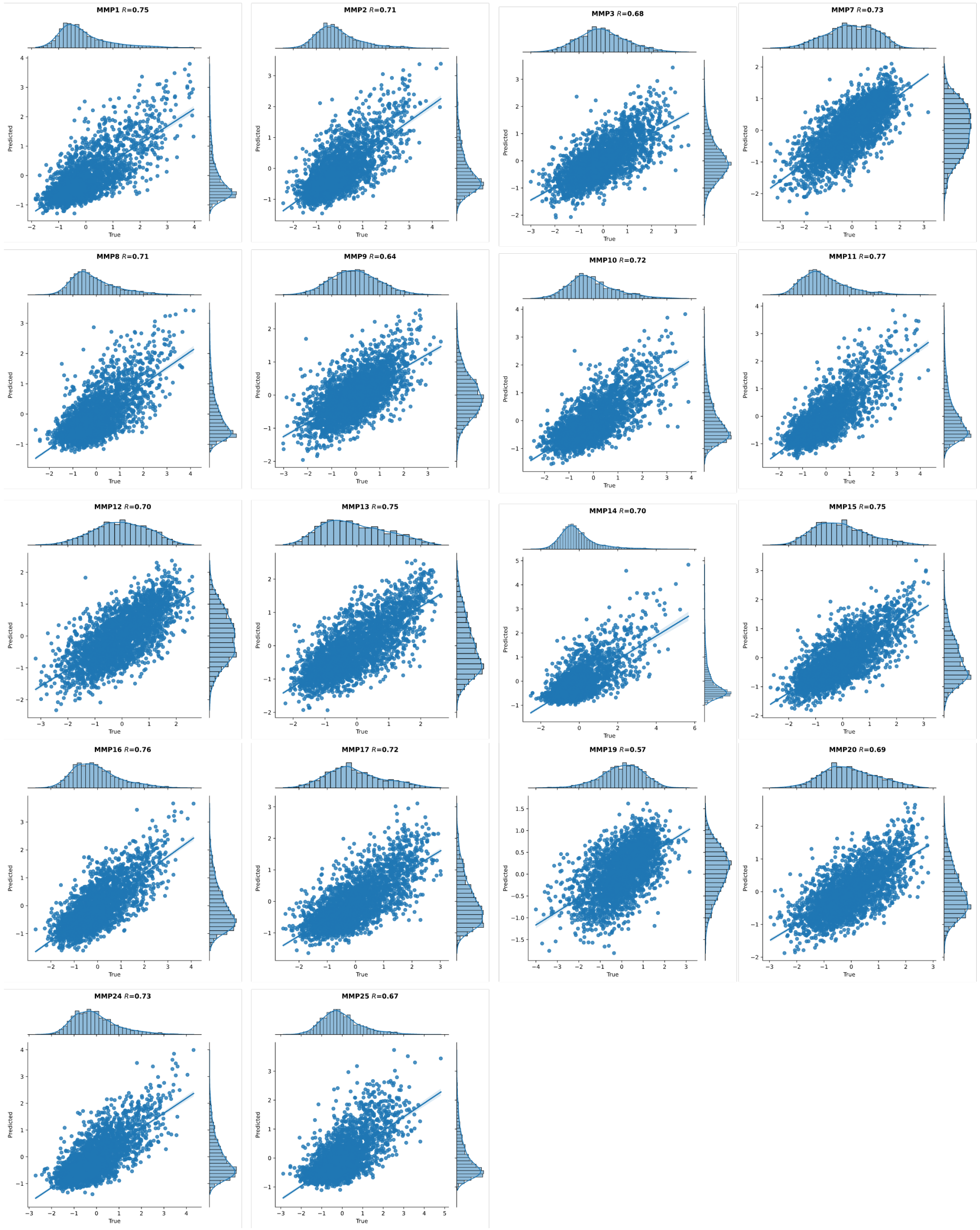

Supplementary Figure 1: **Performance of the LSTM model on Z-score prediction over the mRNA-display test set for each MMP.** The correlation coefficient (Pearson's r) is denoted for each plot.

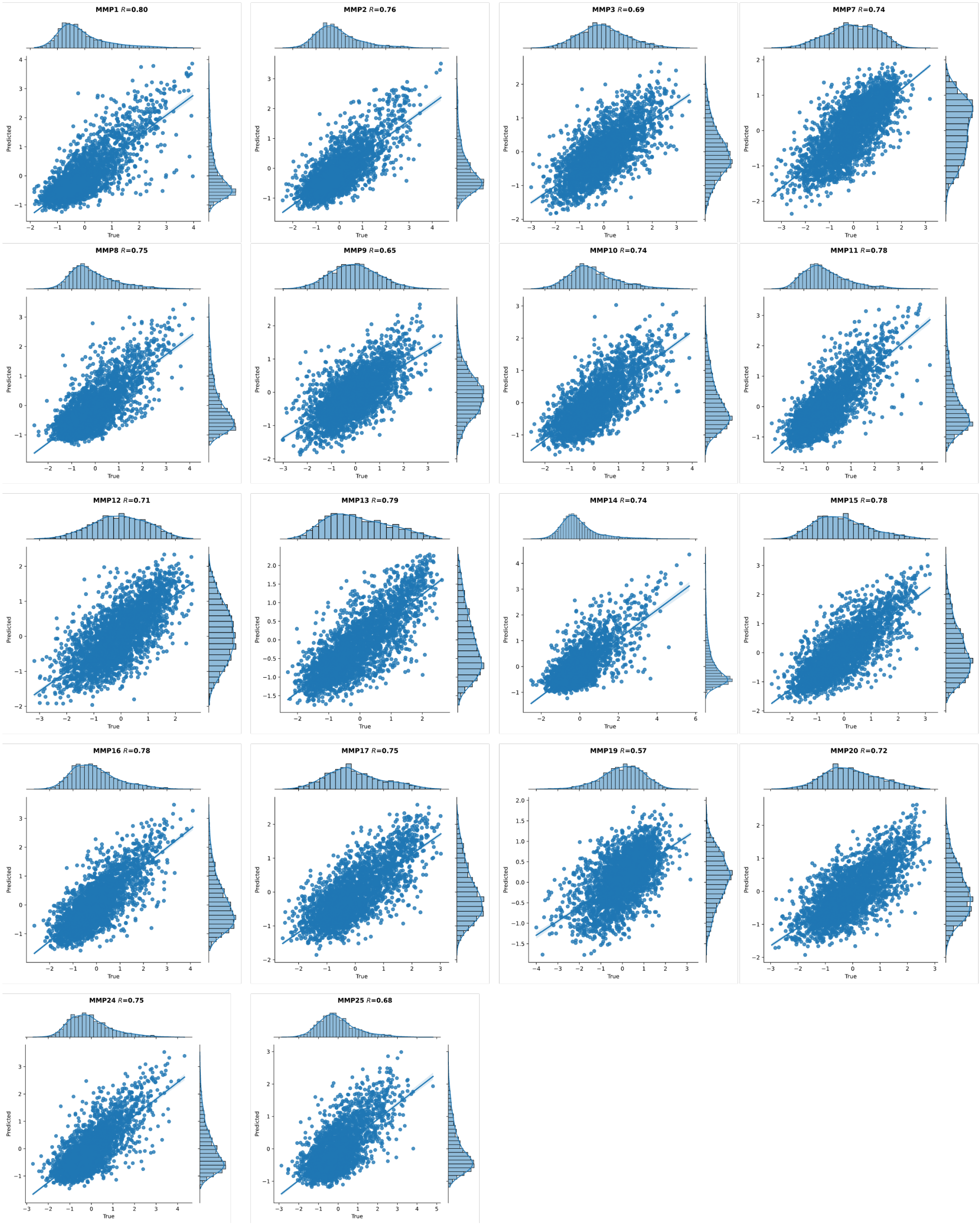

Supplementary Figure 2: **Performance of the Transformer model on Z-score prediction over the mRNA-display test set for each MMP.** The correlation coefficient (Pearson's r) is denoted for each plot.

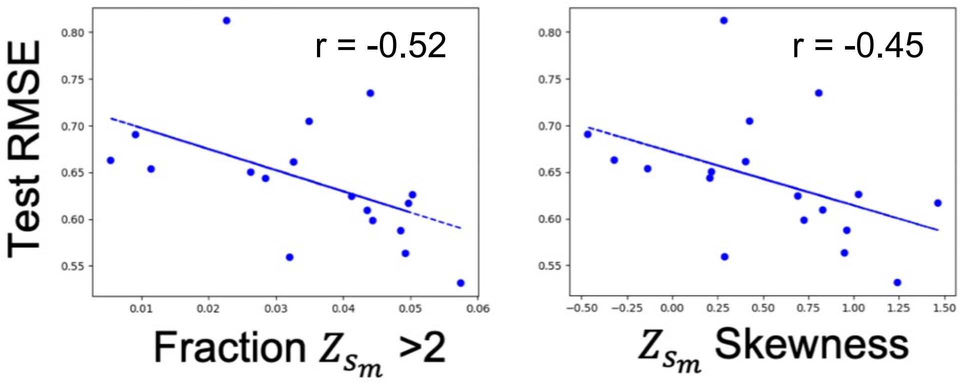

Supplementary Figure 3: **Model performance varies as a function of training cleavage profiles across MMPs.** The fraction of training substrates with high Z-scores and the training distribution skewness both negatively correlate with the RMSE obtained on the test set (Pearson's *r*).

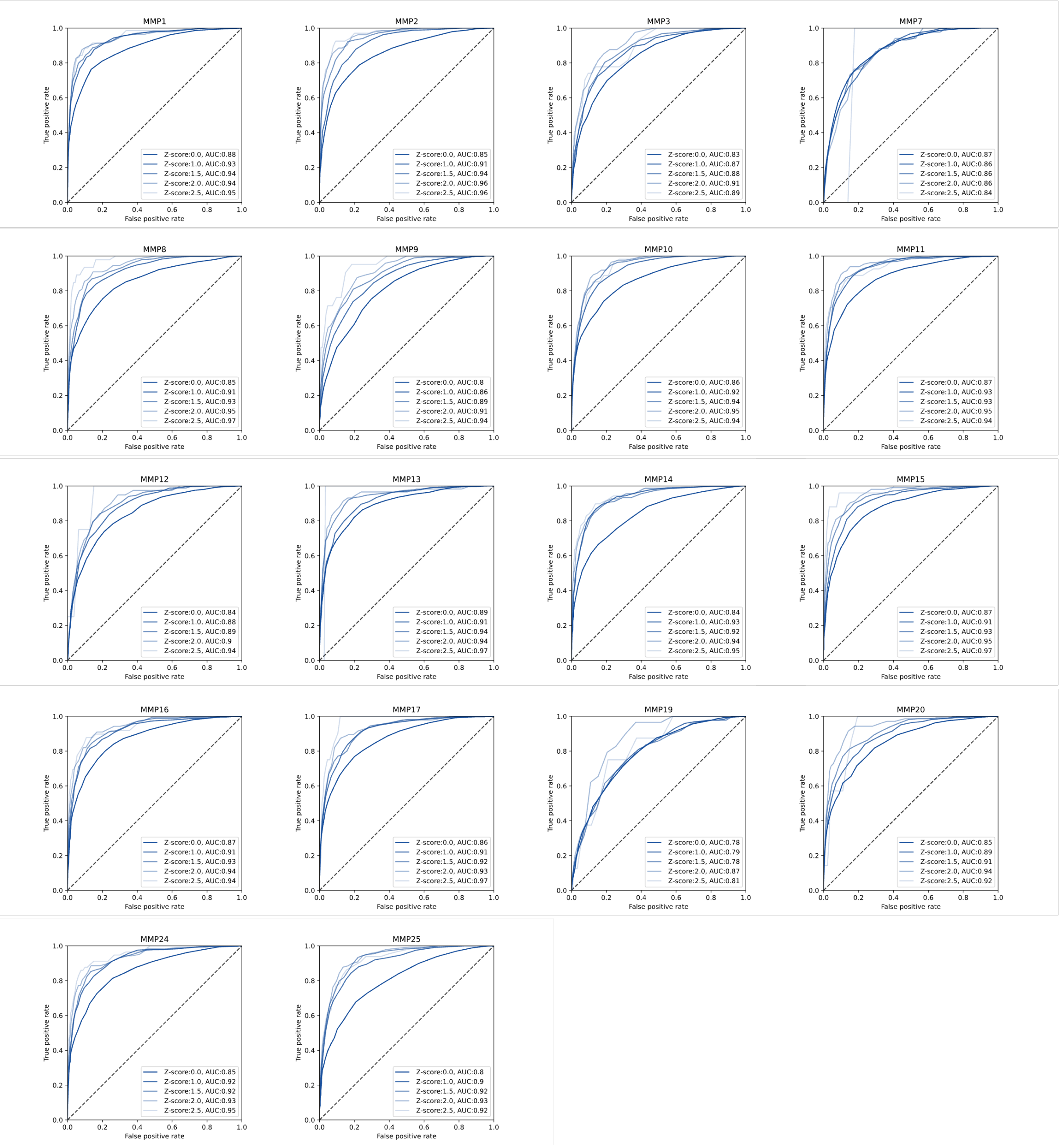

Supplementary Figure 4: **ROC-AUC plot evaluating the transformer predictor over the mRNA-display test set for each MMP.** Individual lines represent performance for different Z-score thresholds, with AUCs provided.

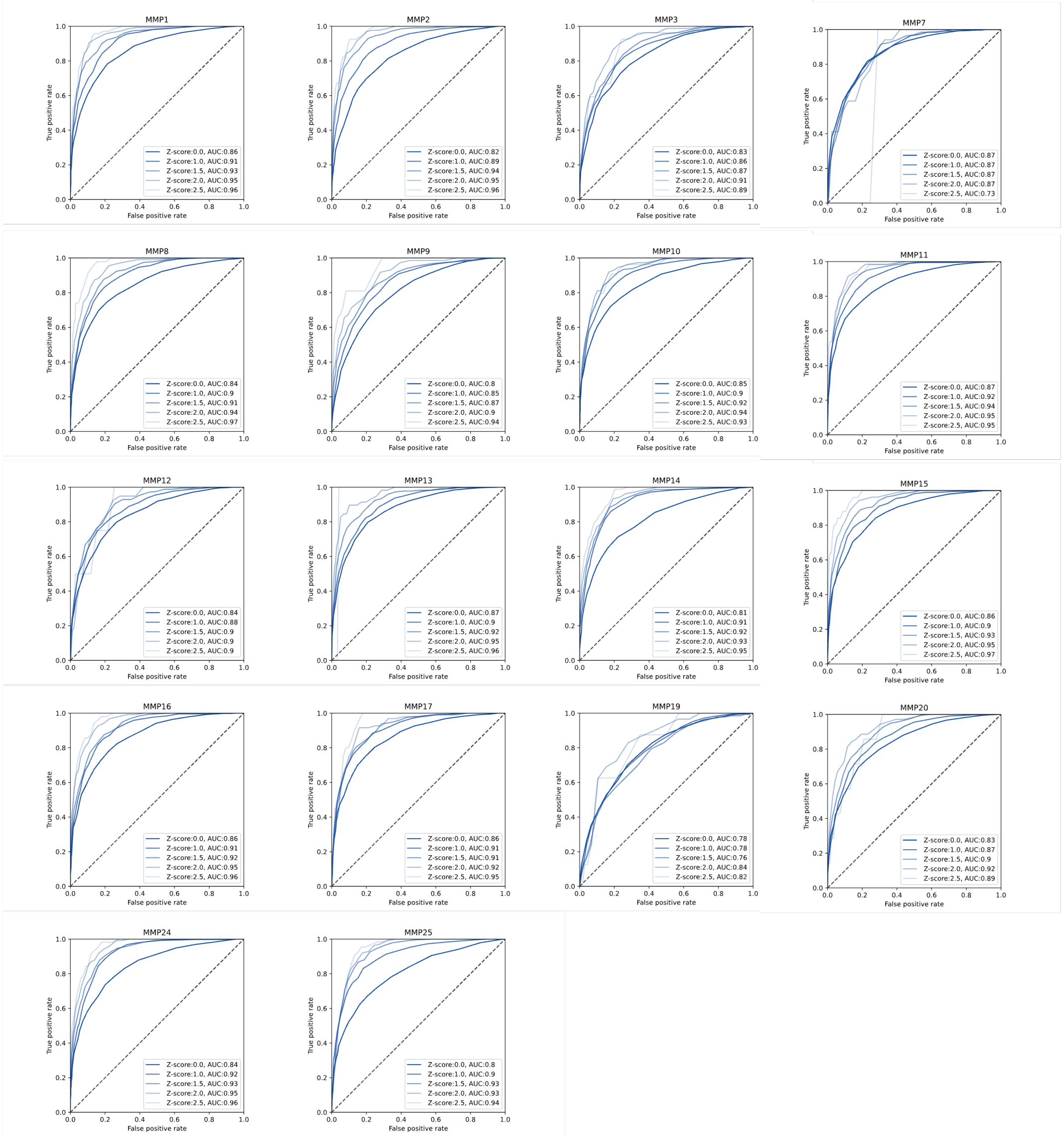

Supplementary Figure 5: **ROC-AUC plot evaluating the LSTM predictor over the mRNA-display test set for each MMP.** Individual lines represent performance for different Z-score thresholds, with AUCs provided.

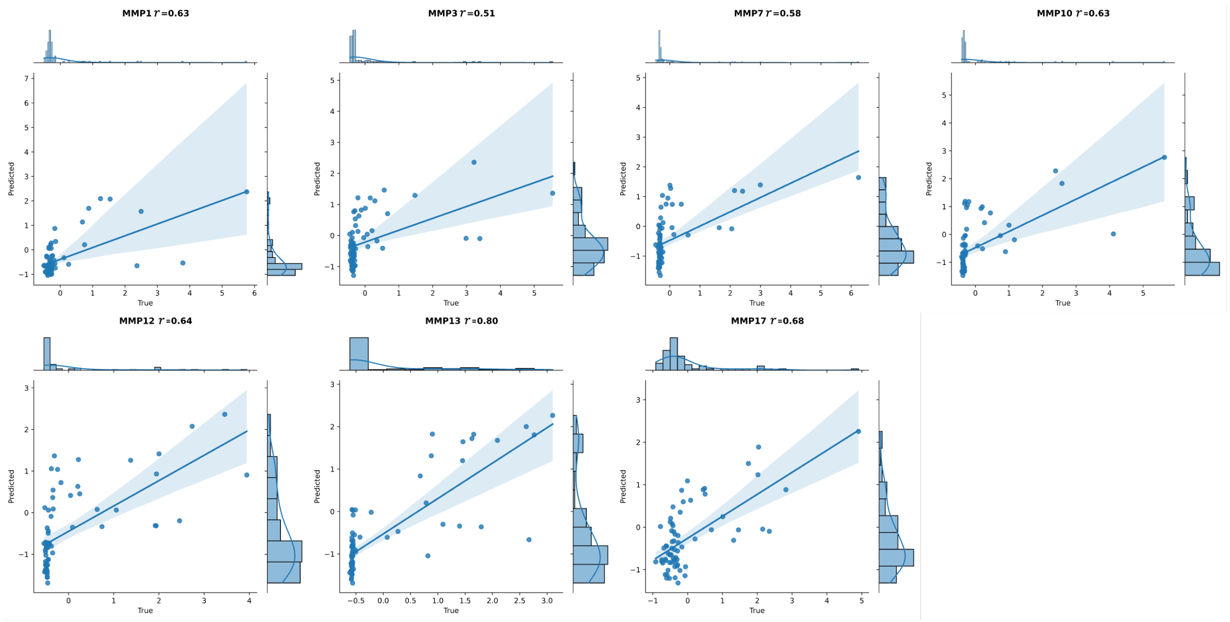

Supplementary Figure 6: **Performance of the transformer model on Z-score prediction over the fluorescence test set for each MMP.** The correlation coefficient (Pearson's r) is denoted for each plot.

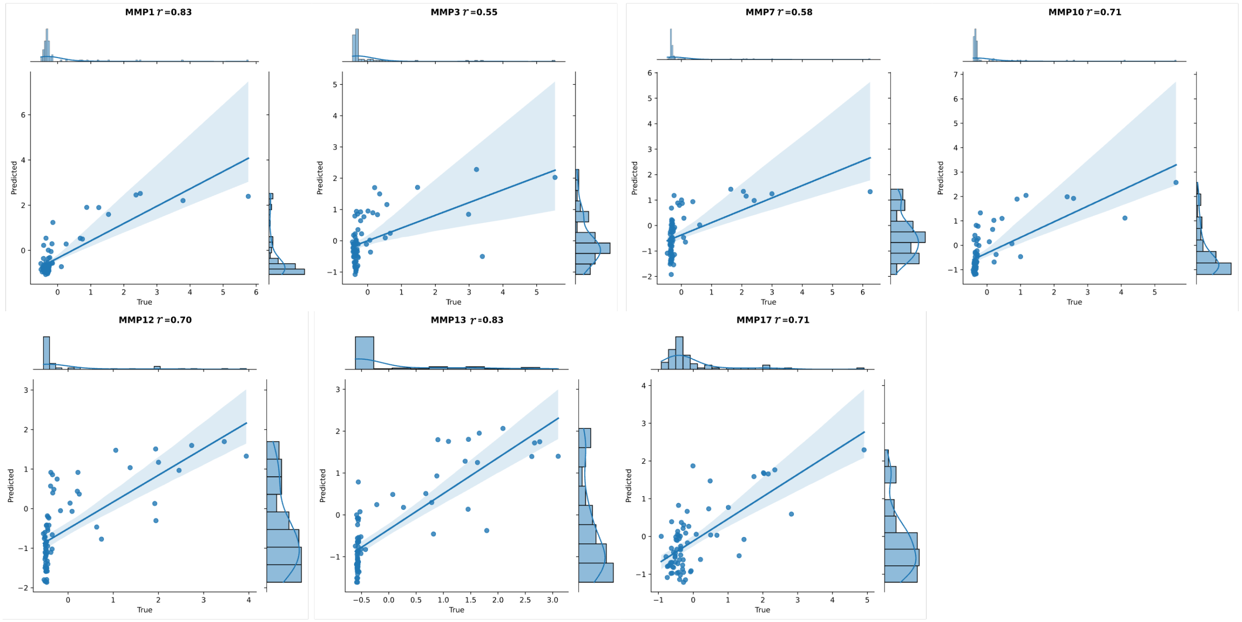

Supplementary Figure 7: **Performance of the LSTM model on Z-score prediction over the fluorescence test set for each MMP.** The correlation coefficient (Pearson's r) is denoted for each plot.

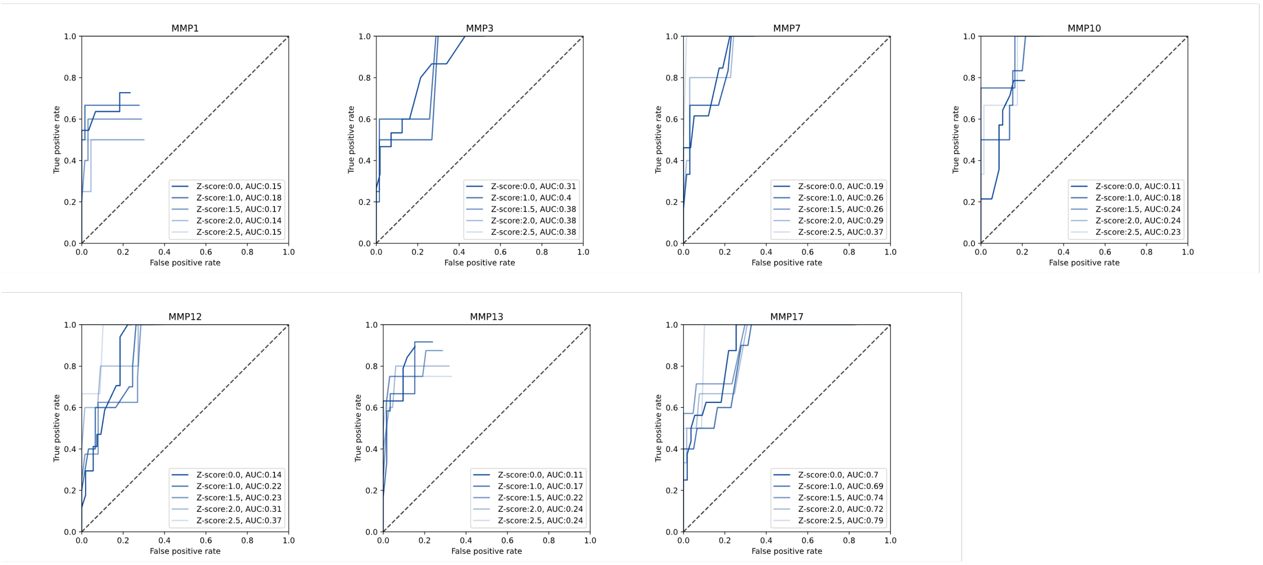

Supplementary Figure 8: **ROC-AUC plot evaluating the transformer predictor over the fluorescence test set for each MMP.** Individual lines represent performance for different Z-score thresholds, with AUCs provided.

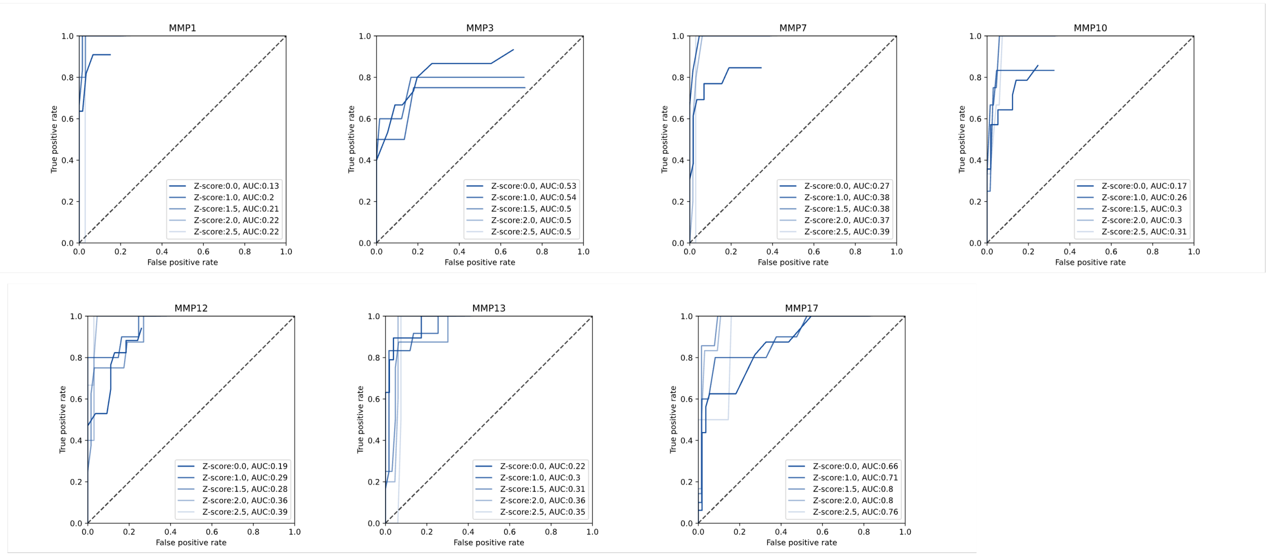

Supplementary Figure 9: **ROC-AUC plot evaluating the LSTM predictor over the fluorescence test set for each MMP.** Individual lines represent performance for different Z-score thresholds, with AUCs provided.

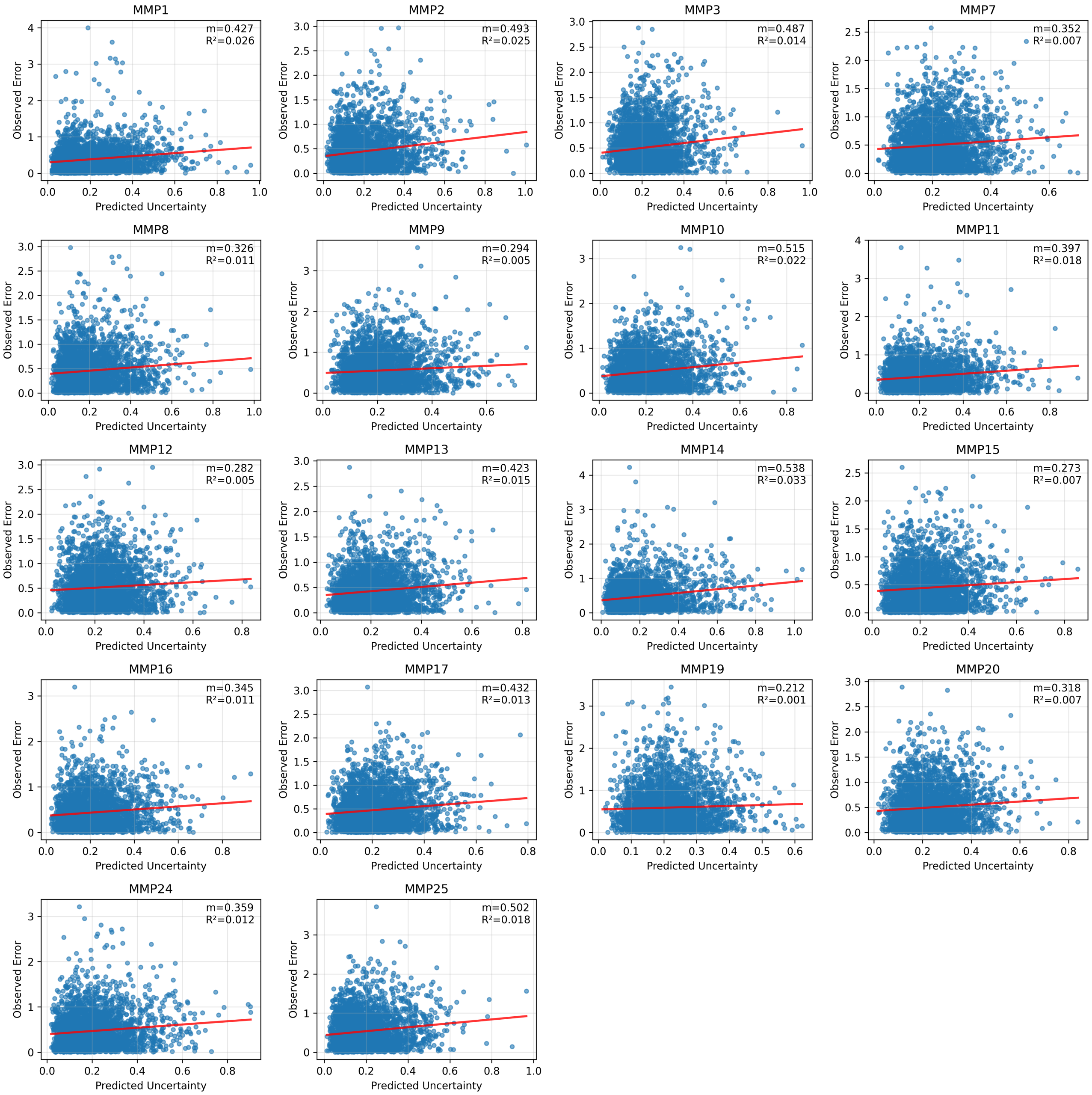

Supplementary Figure 10: **Uncertainty versus observed absolute error for CleaveNet Predictor on the mRNA-display test set sequences and across MMPs.**

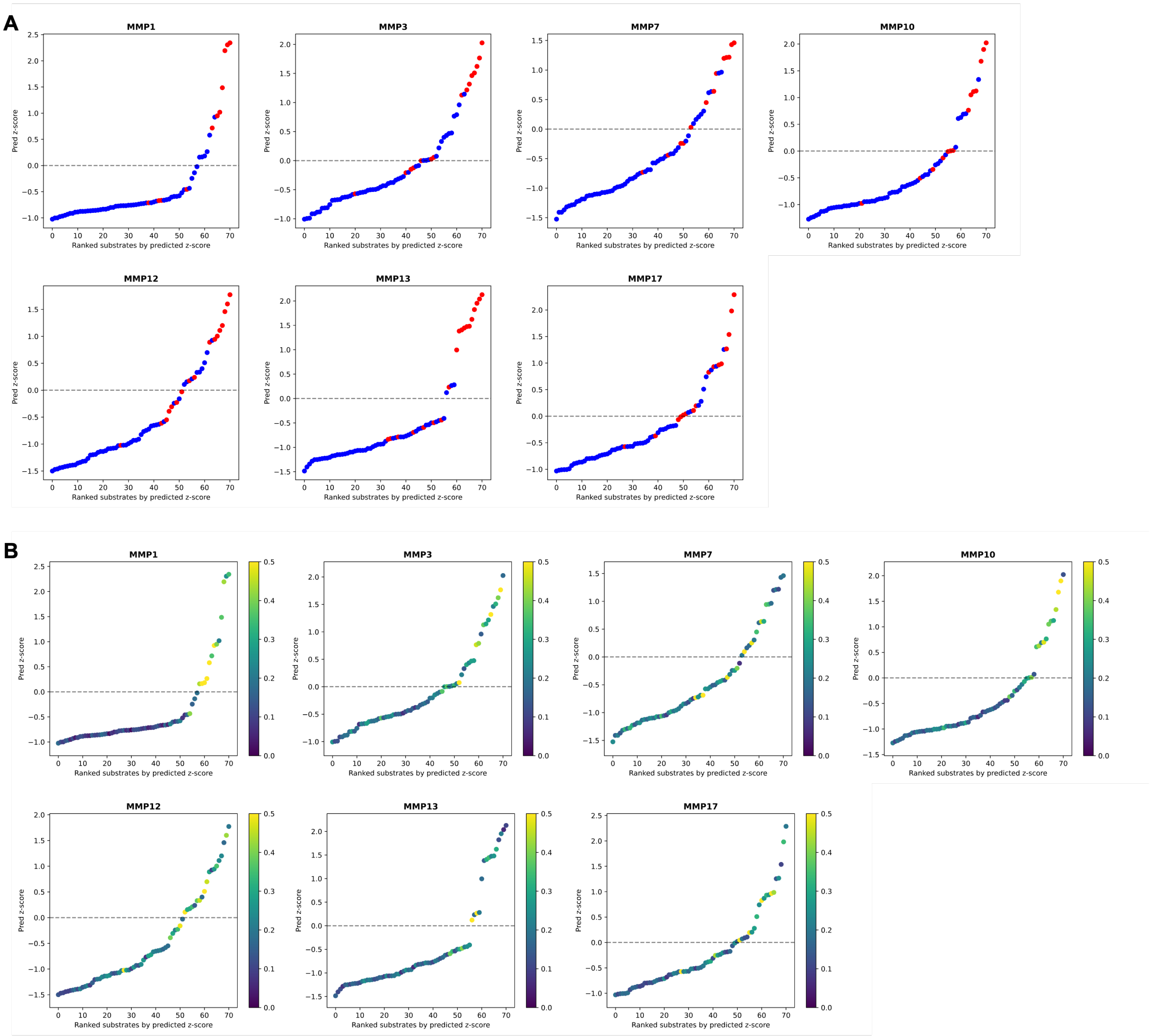

Supplementary Figure 11: **Rank-ordered prediction scores for the transformer predictor, evaluated over the fluorescence test set substrates for each MMP.** (**A**) Each substrate is colored by true cleavage with Z_t=0, where red are cleaved substrates and blue are not-cleaved substrates. Dotted line is at a predicted Z-score of 0. (**B**) Each substrate is colored by the predicted model uncertainty.

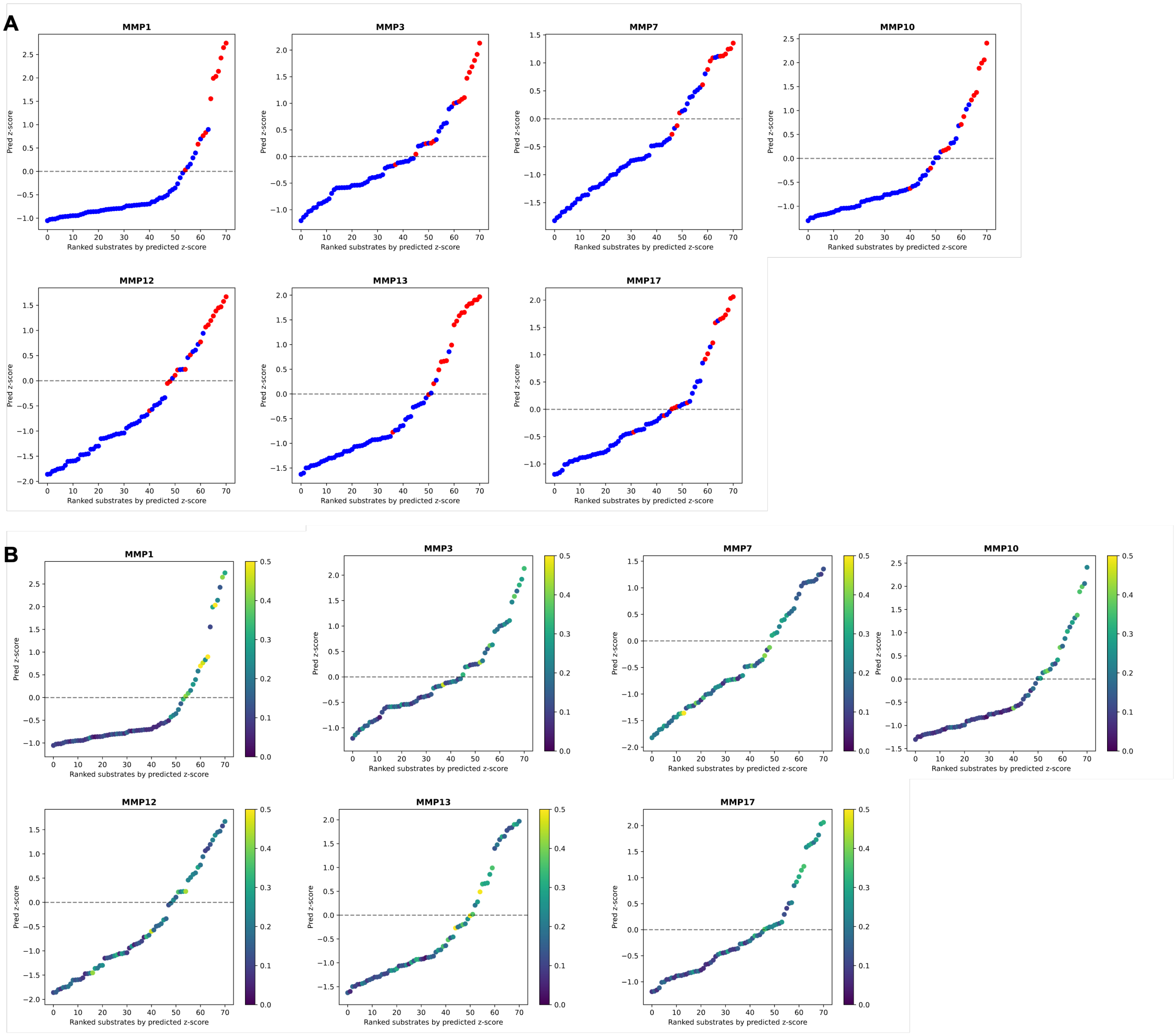

Supplementary Figure 12: **Rank-ordered prediction scores for the LSTM predictor, evaluated over the fluorescence test set substrates for each MMP.** (**A**) Each substrate is colored by true cleavage with Z_t=0, where red are cleaved substrates and blue are not-cleaved substrates. Dotted line is at a predicted Z-score of 0. (**B**) Each substrate is colored by the predicted model uncertainty.

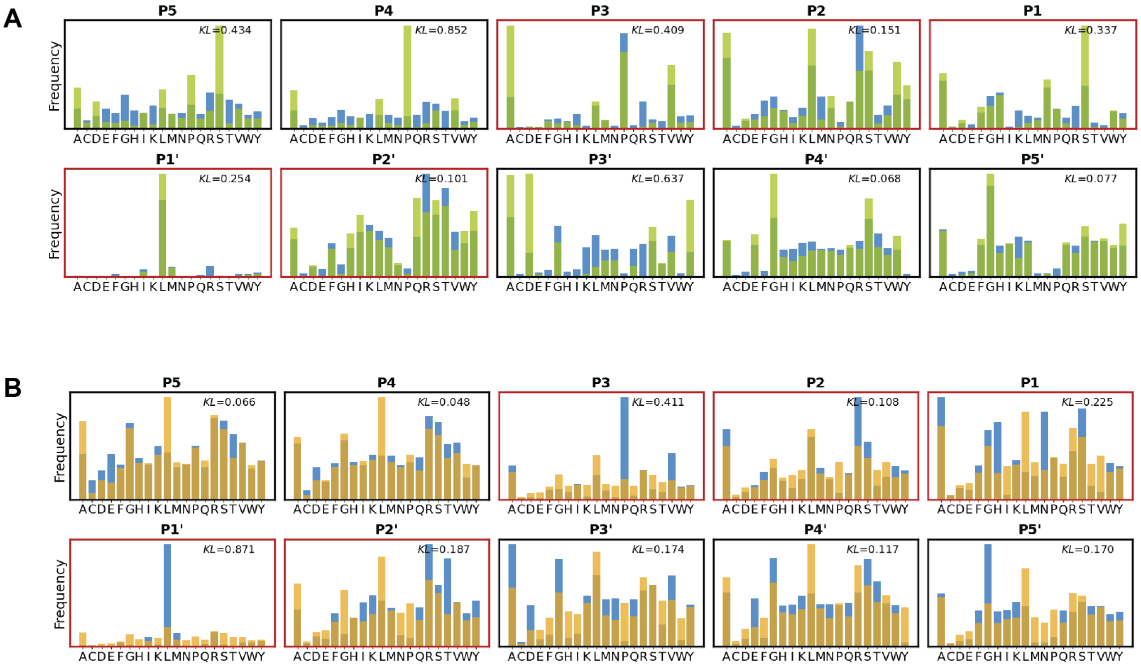

Supplementary Figure 13: **Position-wise amino acid distributions** of (**A**) generated (green, n=4,000) and (**B**) site-independent baseline (yellow, n=4,000) sequences compared to sequences from the mRNA-display test dataset (blue, n=3,717), with the position-wise KL between the CleaveNet-generated or site-independent baseline distributions and the test distribution denoted. Red box outlines indicate positions in the canonical MMP cleavage site.

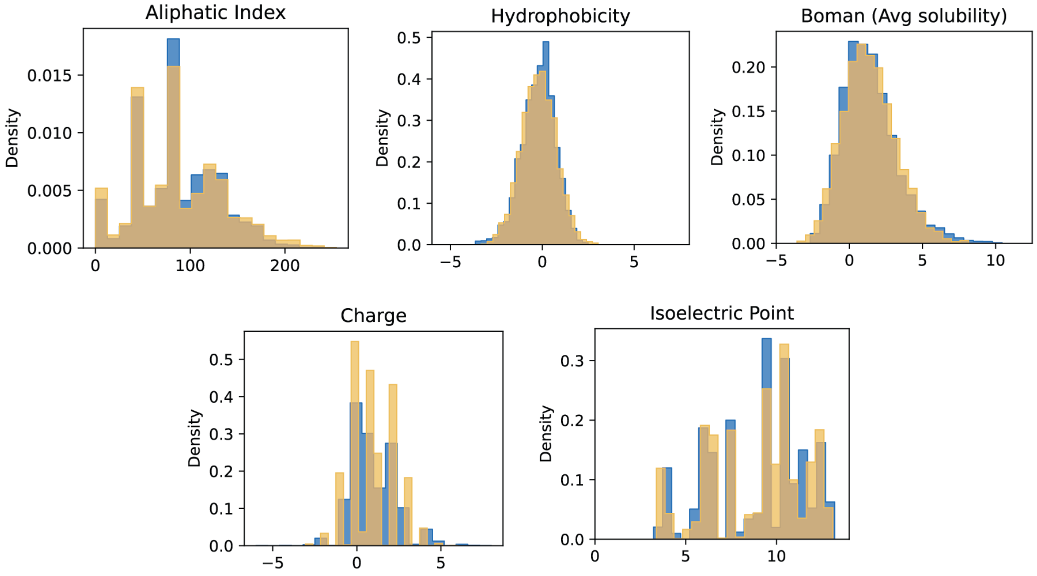

Supplementary Figure 14: **Sequence-level biophysical properties** measured for sequences from the site-independent baseline (yellow, n=4,000) against the mRNA-display test sequences (blue, n=3,717).

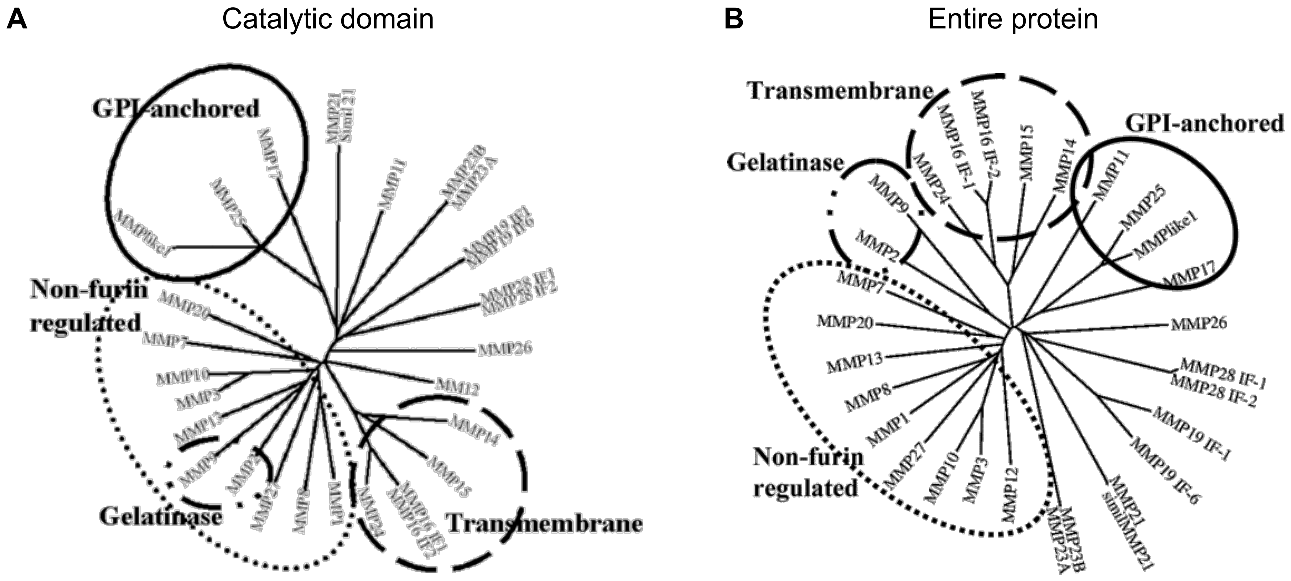

Supplementary Figure 15: **Phylogenetic tree of (A) catalytic domain sequences and (B) entire MMP sequences**. Solid line: GPI-anchored MMPs; broken line: trans-membrane MMPs; line-dotted: gelatinases; dotted: non furin regulated MMPs. Modified from Andreini et al. [58].

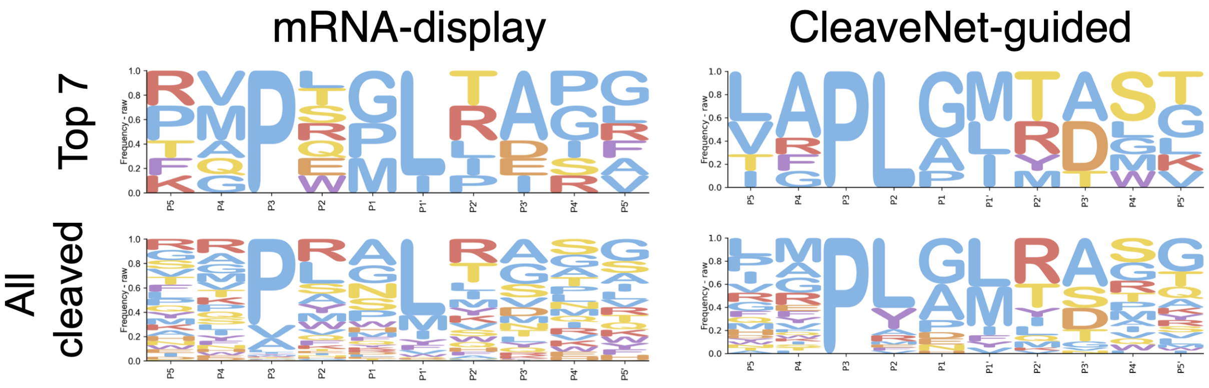

Supplementary Figure 16: **IceLogos of all MMP13 cleavable (bottom) and top-7 most efficiently cleaved (top) substrates** in the mRNA-display training set (left) versus the CleaveNet-guided designed substrates tested *in vitro* (right). Raw amino acid frequencies are displayed.

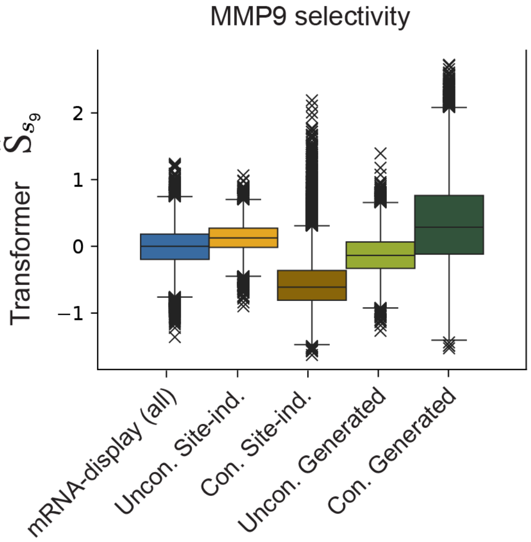

Supplementary Figure 17: **Evaluation of substrates conditionally generated for high MMP9 selectivity**. Predicted MMP9-selectivity scores, $\hat{S}_{s_9}$, for sequences conditionally generated by CleaveNet for high MMP9 selectivity (n=20,000, dark green), relative to sequences from the mRNA-display dataset (n=18,583, blue), unconditional generations (n=19,905, light green), and unconditional and conditional site-independent baselines (n=20,000 each, light and dark yellow, respectively). All pairwise comparisons are significant ($p < 0.0001$) via Kruskal-Wallis test.

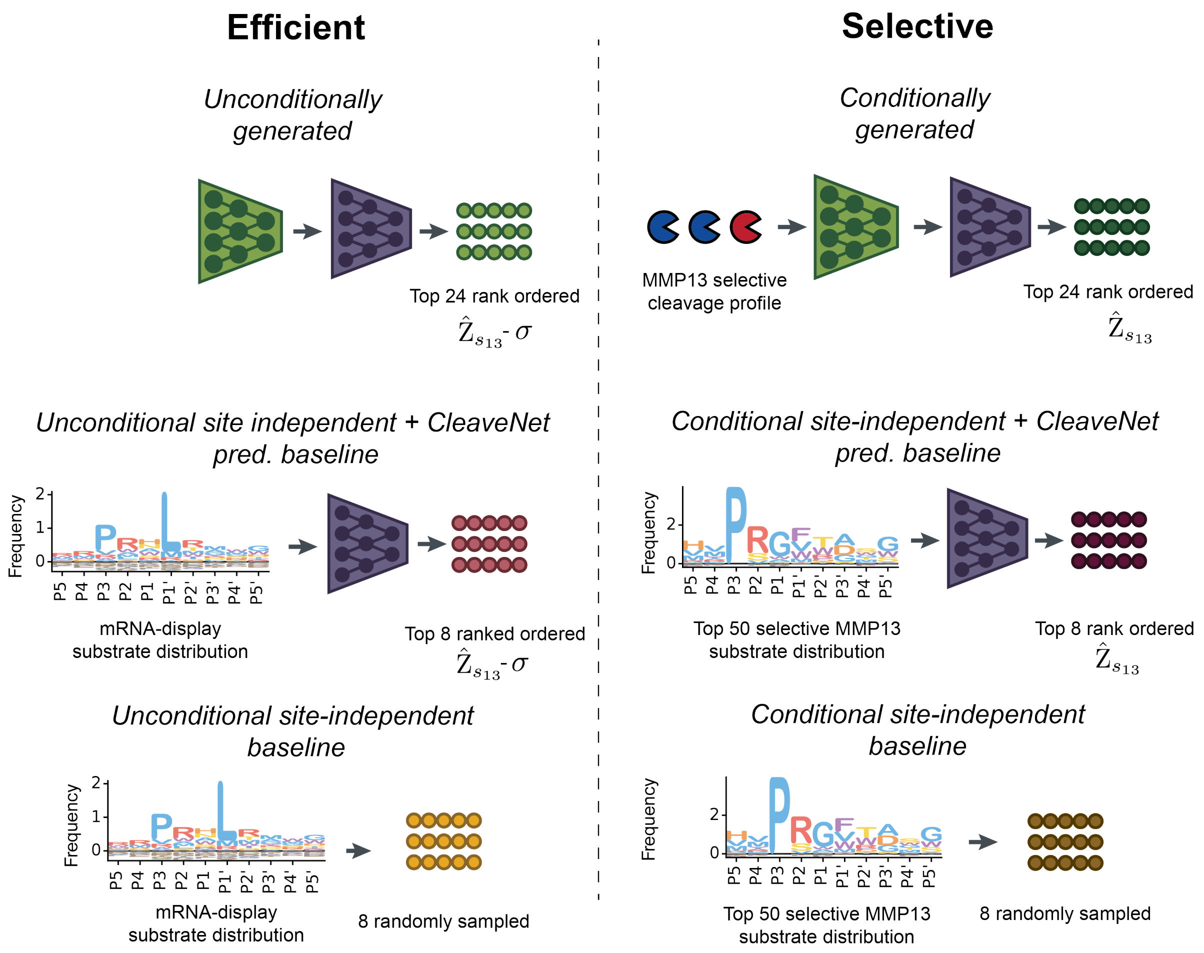

Supplementary Figure 18: **Schematic overview of substrates screened *in vitro*.** Substrates were selected from unconditional generations for efficient cleavage by MMP13 (left), or from conditional generations designed to be cleaved selectively by MMP13 (right). In addition to CleaveNet-generated substrates (green, n=24 per group), appropriate baselines consisting of site-independent baseline only (yellow, n=8 per group) and site-independent baseline + CleaveNet Predictor (burgundy, n=8 per group) were added. Controls from the mRNA-display training set corresponding to substrates that were efficiently, selectively, and not cleaved by MMP13 (not shown, n=5 per group) were also included.

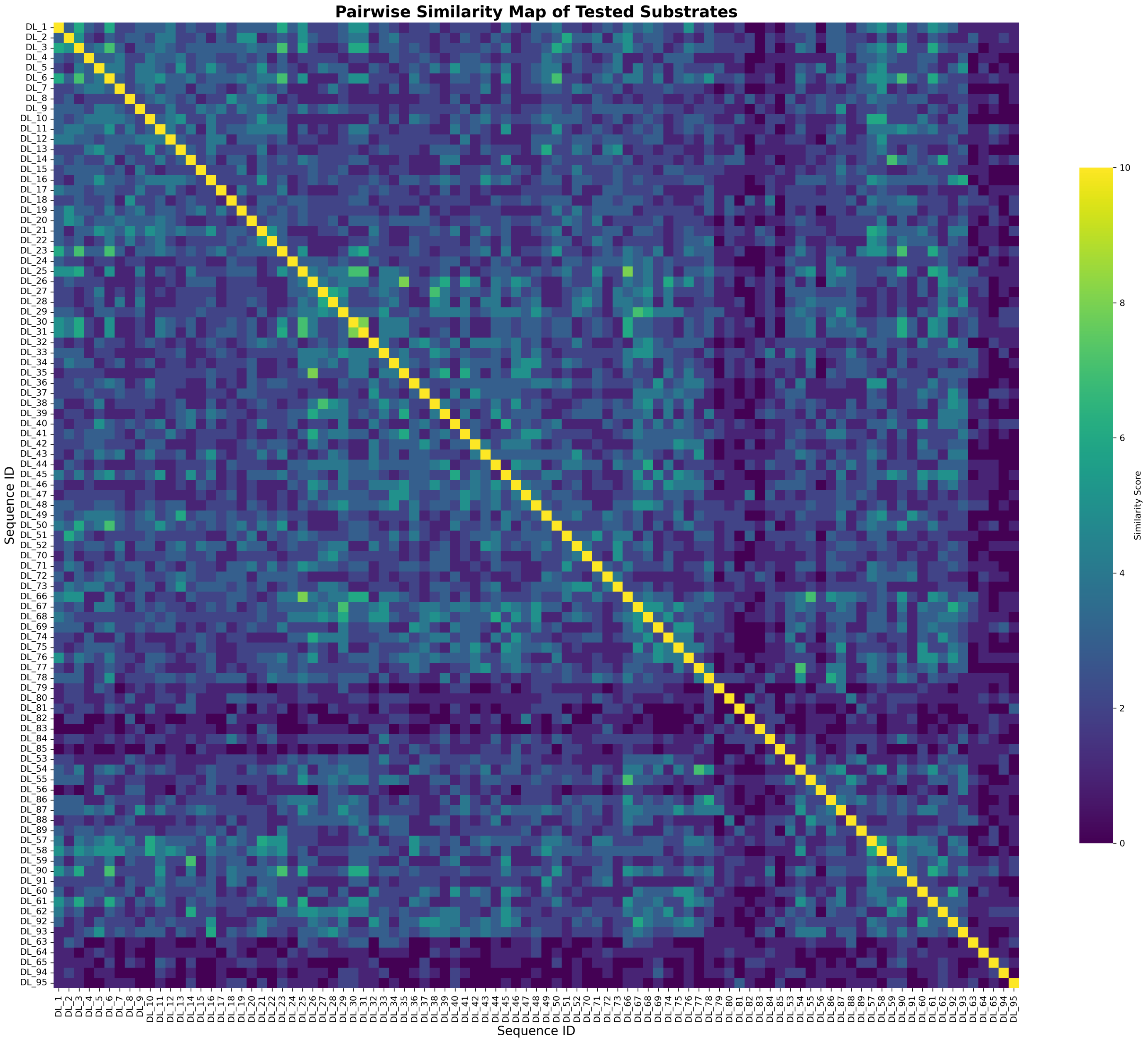

Supplementary Figure 19: **Pairwise similarity map of the 95 substrates tested *in vitro*.** Heatmap showing pairwise sequence similarities calculated using Levenshtein distance on a 0-10 scale. Similarity is measured as Levenshtein distance subtracted from the sequence length. A value of 10 represents identical sequences and a value of 0 represents no shared residues between each pair. Each axis plots the 95 substrate sequences, colored by similarity scores.

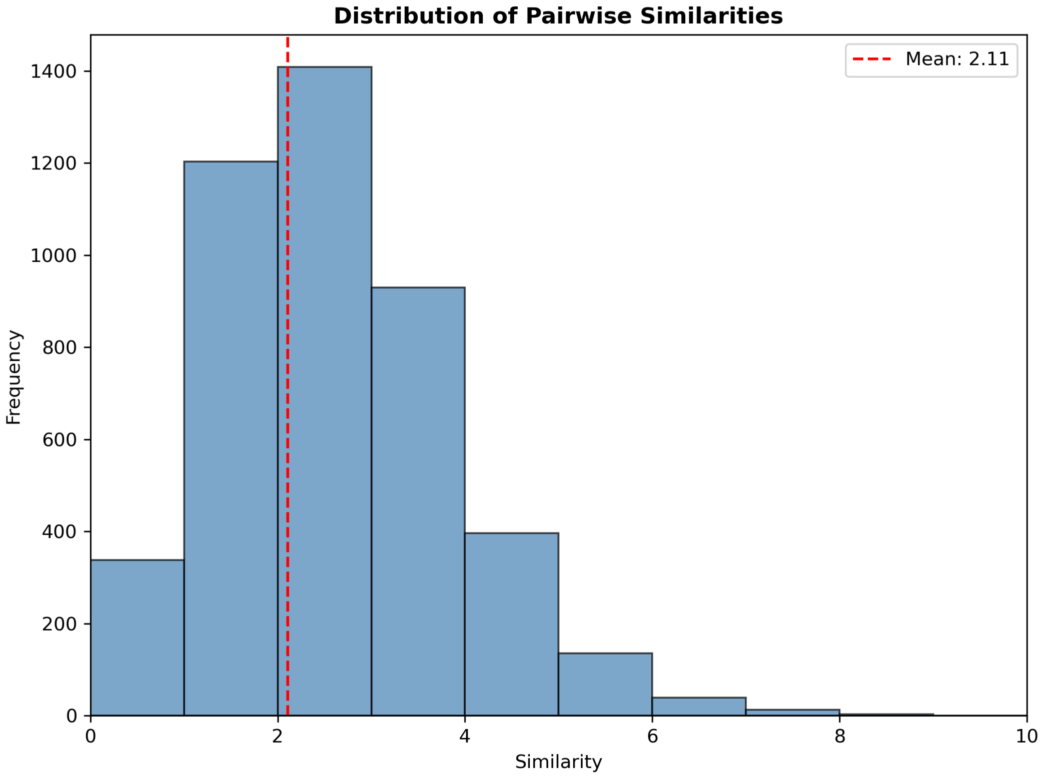

Supplementary Figure 20: **Distribution of pairwise sequence similarities of the 95 substrates tested *in vitro*.** Histogram showing the frequency distribution of similarity scores for all unique pairwise comparisons among the 95 substrates tested *in vitro*. The red dashed line indicates the mean similarity (2.11).

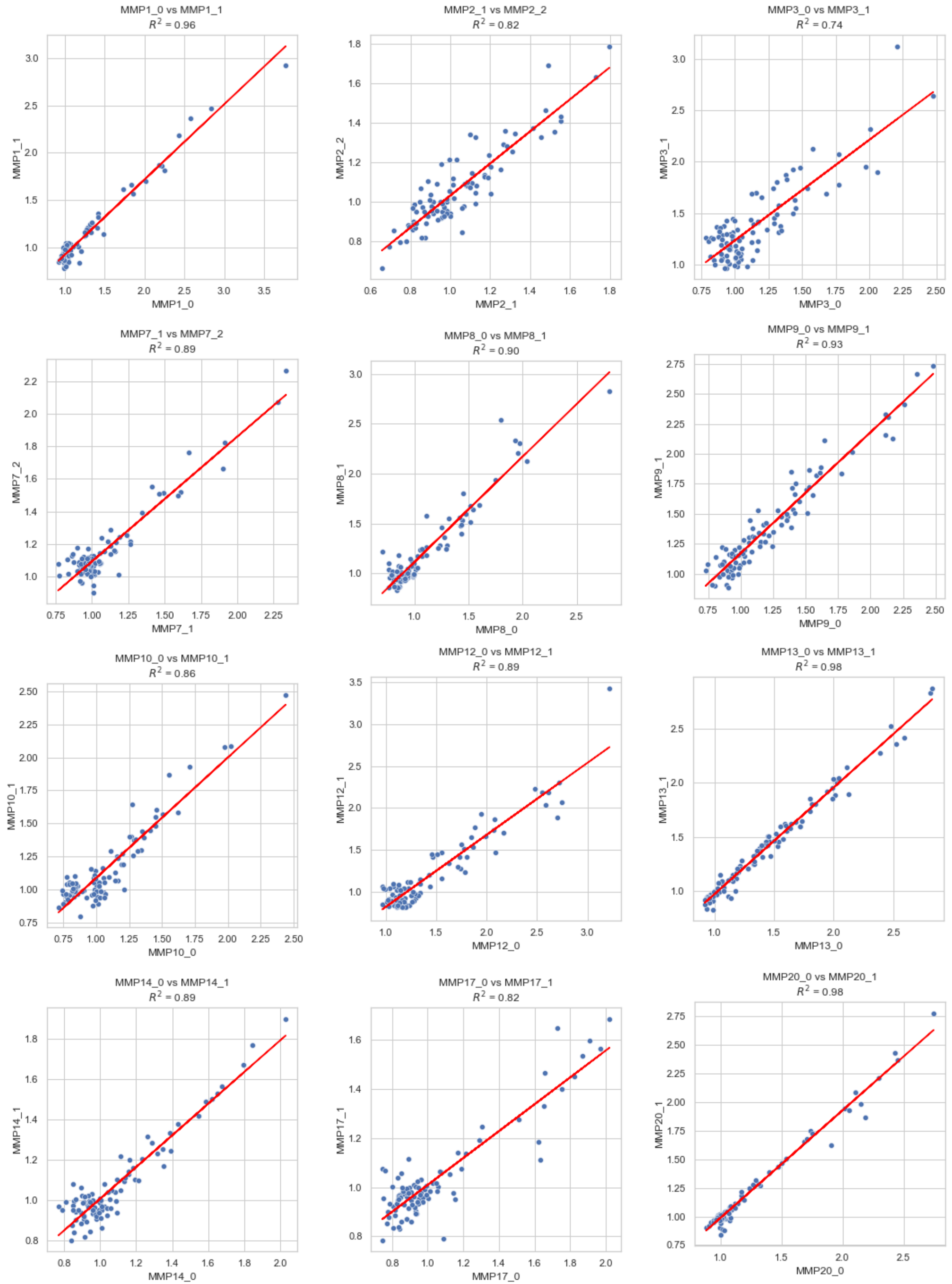

Supplementary Figure 21: **Scatter plots of cleavage rates between technical MMP replicates in the *in vitro* screen.** R^2 denotes the correlation coefficient between replicates, which range between 0.73 for MMP3 and 0.98 for MMP13. Higher correlation is achieved for cleaved substrates above the noise floor of the assay.

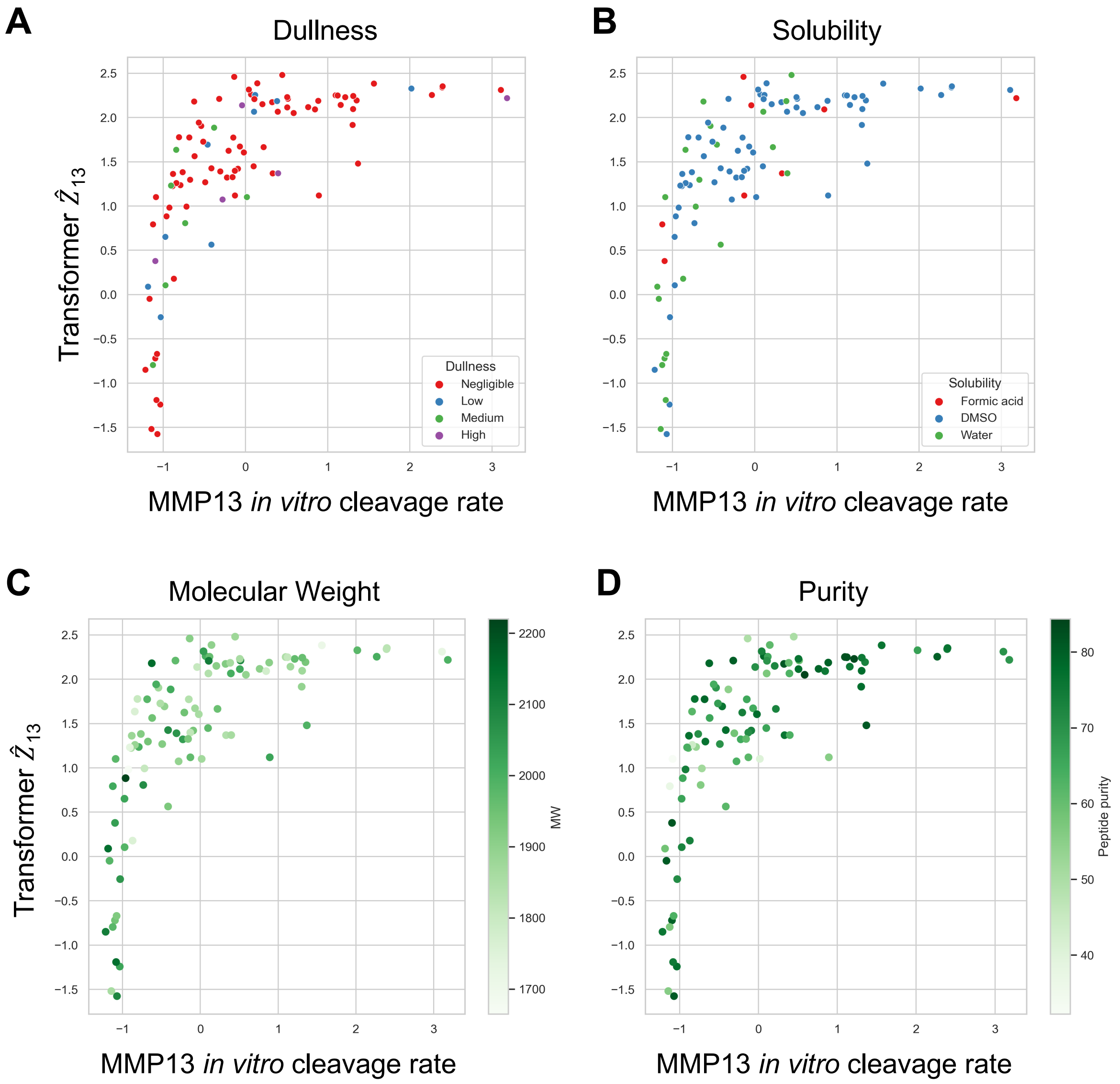

Supplementary Figure 22: **Scatter plots of *in vitro* MMP13 cleavage rates vs. CleaveNet-predicted cleavage scores, color-coded by peptide properties.** Crude peptides were (**A**) manually annotated by their perceived dullness (negligible, low, medium and high), and manufacturer-reported (**B**) solubility, (**C**) molecular weight, and (**D**) purity.

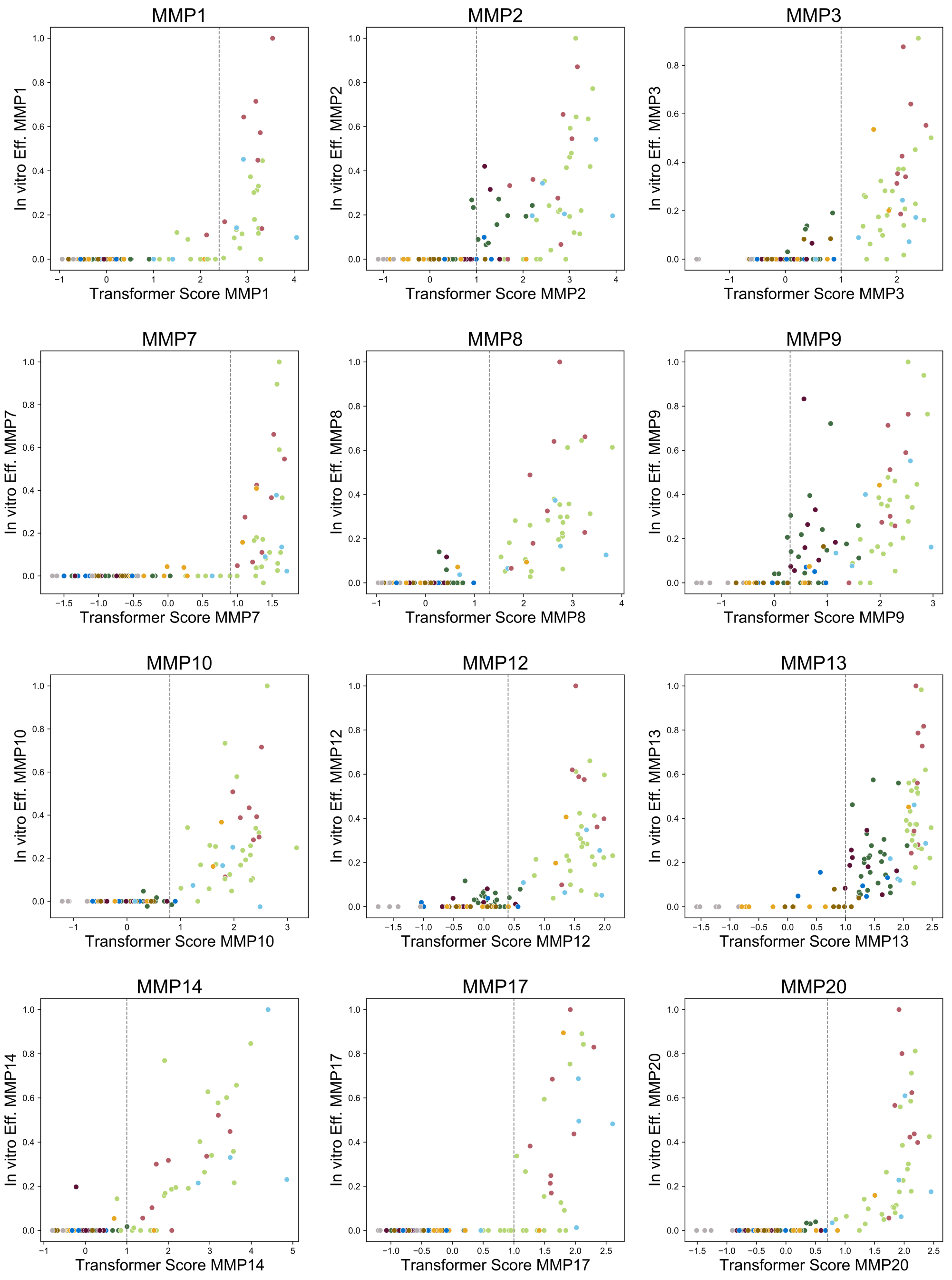

Supplementary Figure 23: **Scatter plots of CleaveNet predicted scores (with Transformer) vs. *in vitro* efficiency scores for all substrates in the screen across MMPs.** Substrates are color-coded by group and cleavage thresholds in Supplementary Table 6 denoted by vertical dotted lines.

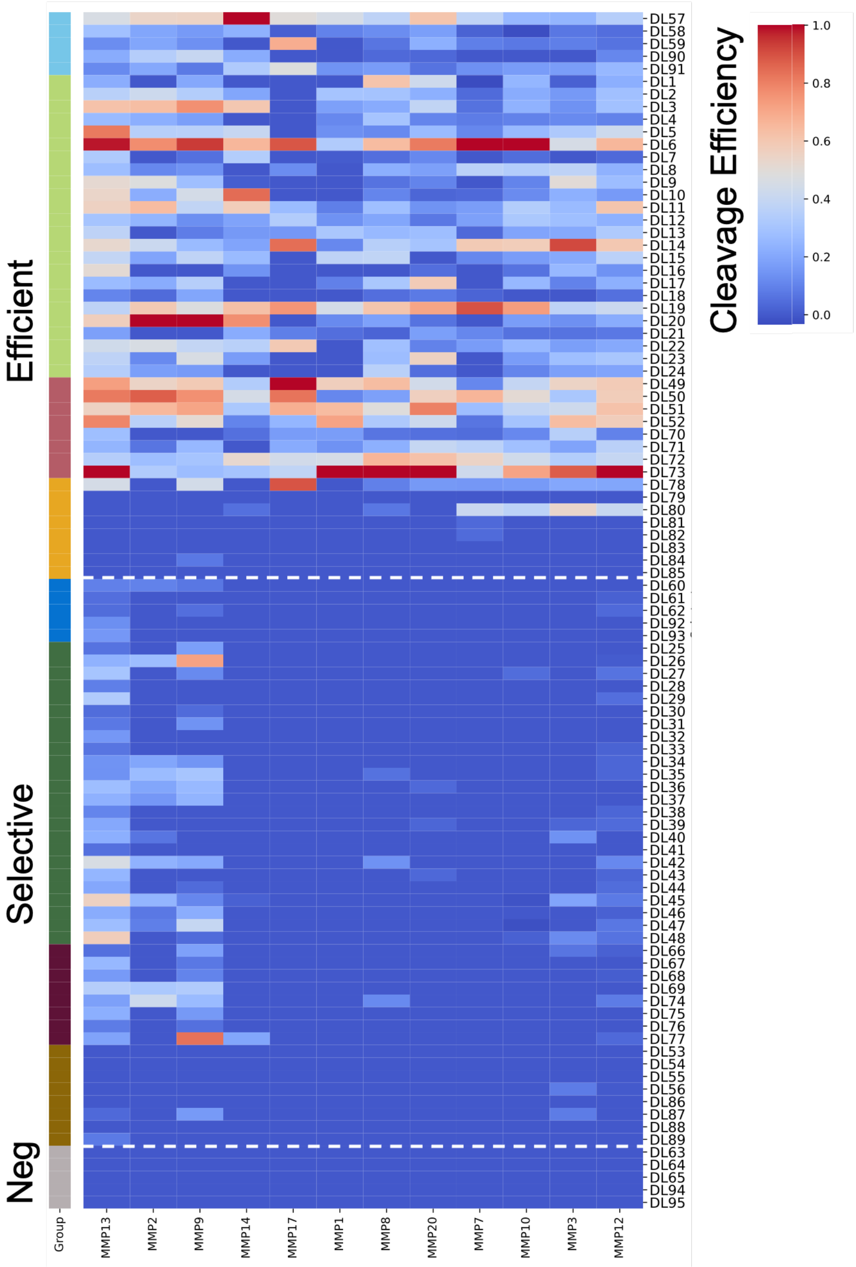

Supplementary Figure 24: **Heatmap showing *in vitro* cleavage efficiencies for all substrate-protease pairs.** Substrates were ordered by their expected MMP13 cleavage profile: efficient (top), selective (middle), uncleaved (bottom). In addition to CleaveNet-generated substrates (green, n=24 per group), appropriate baselines consisting of site-independent baseline alone (yellow, n=8 per group) and site-independent + CleaveNet Predictor (burgundy, n=8 per group) were added. Controls from the mRNA-display training set corresponding to substrates that were efficiently, selectively, or not cleaved by MMP13 (light blue, dark blue, and grey respectively, n=5 per group) were also included.

sequence	MMP1	MMP2	MMP3	MMP7	MMP8	MMP9	MMP10	MMP11	MMP12	MMP13	MMP14	MMP15	MMP16	MMP17	MMP19	MMP20	MMP24	MMP25
LAAYRLEDTF	0.11	-0.52	0.18	1.28	0.07	0.66	0.91	-0.15	0.53	-0.05	-0.47	-0.11	-1.24	0.67	0.53	1.35	-0.38	0.55
KQARLIMQAI	0.2	0.36	1	1.34	0.13	1.59	0.27	0.17	0.54	-0.41	-0.38	-0.29	-0.44	0.85	1.66	0.51	-0.48	0.73
AARPGFGLSP	-0.85	-0.37	-0.73	-1.19	-1.26	-0.5	-1.32	-1.53	-1.92	-0.44	-0.68	-1.29	-0.94	-1.28	-0.65	-0.76	-0.99	-0.5
LKAYKSELEE	-0.57	-0.11	-0.12	0.6	-0.41	0.66	-0.9	-0.49	0.32	-0.38	-1.22	-0.93	-0.99	-0.44	-0.36	0.72	-0.86	-0.67
RTRDRLDEVK	0	-1.19	-0.46	-0.25	-0.05	-0.36	-0.18	-0.86	-0.32	-0.34	-0.28	-0.12	-0.71	0.04	0.83	0.02	-0.28	-0.08
RQWAGLVEKV	0.25	0.19	0.1	1.15	-0.23	0.49	-0.49	0.15	-0.03	-0.7	1.84	1.59	1.39	-0.23	0.63	0.86	1.12	-0.19
KGEYRTNPED	-0.87	-1.19	-1.13	-0.82	-0.62	-0.98	-0.54	-0.49	0.23	-1.43	-0.38	-0.42	-0.72	-0.03	-0.7	0.27	-0.71	-0.48
STSGGYIFYT	-1.25	-0.07	-0.32	-0.6	-0.37	-0.46	-0.82	-0.6	-0.94	-1.16	-0.22	-0.28	-0.28	-0.96	0	-1.31	-0.34	0.25
HIDDKAFENV	-0.47	-0.18	0.14	-0.06	-0.43	-0.71	-0.47	-0.87	-0.39	-0.61	-0.31	-1.03	-0.83	-0.57	0.07	0.1	-0.83	-0.51
TVNENLENYY	-0.4	0.35	0.4	0	0.76	1.46	-0.36	0.3	1.72	-0.06	0.55	0.83	0.33	0.89	1.99	1.14	0.46	1.84
Threshold	*2.4*	*1.0*	*1.0*	*0.9*	*1.3*	*0.3*	*0.8*	*1.0*	*0.4*	*1.0*	*1.0*	*1.0*	*1.0*	*1.0*	*1.0*	*0.7*	*1.0*	*1.0*

sequence	MMP1	MMP2	MMP3	MMP7	MMP8	MMP9	MMP10	MMP11	MMP12	MMP13	MMP14	MMP15	MMP16	MMP17	MMP19	MMP20	MMP24	MMP25
LAAYRLEDTF	0.00	0.00	0.00	0.17	0.00	0.09	0.03	0.00	0.05	0.00	0.00	0.00	0.00	0.00	0.00	0.26	0.00	0.00
KQARLIMQAI	0.00	0.00	0.00	0.19	0.00	0.33	0.00	0.00	0.05	0.00	0.00	0.00	0.00	0.00	0.29	0.00	0.00	0.00
AARPGFGLSP	0.00	0.00	0.00	0.00	0.00	0.00	0.00	0.00	0.00	0.00	0.00	0.00	0.00	0.00	0.00	0.00	0.00	0.00
LKAYKSELEE	0.00	0.00	0.00	0.00	0.00	0.09	0.00	0.00	0.00	0.00	0.00	0.00	0.00	0.00	0.00	0.01	0.00	0.00
RTRDRLDEVK	0.00	0.00	0.00	0.00	0.00	0.00	0.00	0.00	0.00	0.00	0.00	0.00	0.00	0.00	0.00	0.00	0.00	0.00
RQWAGLVEKV	0.00	0.00	0.00	0.11	0.00	0.05	0.00	0.00	0.00	0.00	0.14	0.23	0.11	0.00	0.00	0.06	0.03	0.00
KGEYRTNPED	0.00	0.00	0.00	0.00	0.00	0.00	0.00	0.00	0.00	0.00	0.00	0.00	0.00	0.00	0.00	0.00	0.00	0.00
STSGGYIFYT	0.00	0.00	0.00	0.00	0.00	0.00	0.00	0.00	0.00	0.00	0.00	0.00	0.00	0.00	0.00	0.00	0.00	0.00
HIDDKAFENV	0.00	0.00	0.00	0.00	0.00	0.00	0.00	0.00	0.00	0.00	0.00	0.00	0.00	0.00	0.00	0.00	0.00	0.00
TVNENLENYY	0.00	0.00	0.00	0.00	0.00	0.30	0.00	0.00	0.48	0.00	0.00	0.00	0.00	0.00	0.43	0.17	0.00	0.20

Supplementary Figure 25: **Comparison between mRNA display Z-scores (top) and corrected efficiencies (bottom) for 10 sequences in the training set** Corrected efficiencies were calculated using the `corrected_efficiency` function that leverages cleavage thresholds inferred from the *in vitro* screening data (top table, last row italics). After transformation to corrected efficiencies, the sequence with the highest cleavage for a given MMP has a value of 1, a fractional efficiency between 0 and 1 is assigned to all other cleaved substrates, and a value of 0 is assigned to non-cleaved substrates. This improves data interpretability, substrate selection, and model conditioning. It also brings all values for all MMPs between 0 and 1, such that relative differences across substrates can be more effectively compared within a given MMP. The `corrected_selectivity` function then streamlines selectivity analysis.

Supplementary Table 1: **Performance of the CleaveNet Predictor.** For each MMP and test set, the average mean absolute error (MAE) and standard deviation over an ensemble of n=5 is provided for each model class.

Protease	Transformer mRNA-display Test	LSTM mRNA-display Test	Transformer Fluorescence Test	LSTM Fluorescence Test
MMP1	0.421 ± 0.006	0.452 ± 0.012	0.636 ± 0.058	0.513 ± 0.045
MMP2	0.478 ± 0.007	0.518 ± 0.002	-	-
MMP3	0.546 ± 0.007	0.561 ± 0.002	0.593 ± 0.034	0.601 ± 0.041
MMP7	0.534 ± 0.008	0.545 ± 0.002	0.764 ± 0.093	0.808 ± 0.089
MMP8	0.491 ± 0.006	0.519 ± 0.009	-	-
MMP9	0.583 ± 0.008	0.599 ± 0.003	-	-
MMP10	0.512 ± 0.003	0.529 ± 0.003	0.713 ± 0.050	0.652 ± 0.035
MMP11	0.462 ± 0.005	0.448 ± 0.010	-	-
MMP12	0.554 ± 0.007	0.562 ± 0.009	0.793 ± 0.058	0.801 ± 0.041
MMP13	0.468 ± 0.004	0.514 ± 0.005	0.610 ± 0.055	0.590 ± 0.045
MMP14	0.498 ± 0.007	0.522 ± 0.005	-	-
MMP15	0.488 ± 0.007	0.515 ± 0.002	-	-
MMP16	0.478 ± 0.006	0.502 ± 0.003	-	-
MMP17	0.513 ± 0.006	0.547 ± 0.006	0.595 ± 0.040	0.534 ± 0.046
MMP19	0.620 ± 0.004	0.629 ± 0.002	-	-
MMP20	0.538 ± 0.003	0.567 ± 0.008	-	-
MMP24	0.504 ± 0.005	0.526 ± 0.003	-	-
MMP25	0.560 ± 0.008	0.579 ± 0.003	-	-

Supplementary Table 2: **CleaveNet generator ablation experiments**. The training task, model parameters, and lowest cross entropy test loss is given for each experiment. The final CleaveNet generator is in bold.

Model	Task	Parameters	Test Loss
LSTM	Unconditional	25k	2.220
LSTM	Unconditional	42k	2.283
Transformer	Unconditional	56k	2.203
Transformer	Unconditional	84k	2.139
Transformer	Conditional	84k	1.998
Transformer	Both	56k	2.196
Transformer	Both	84k	2.130
**Transformer**	Both	328k	1.980

Supplementary Table 3: **Summary statistics of *in vitro* MMP13 cleavage efficiency metrics by group.** "Uncond." and "Cond." represent unconditionally and conditionally, respectively.

Group	Max.	Min.	StdDev	Avg.	Med.	Cleaved
Uncond. Site-independent	0.45	0.00	0.16	0.06	0.00	1/8
Uncond. Site-independent + CleaveNet Pred.	1.00	0.24	0.28	0.59	0.64	8/8
Uncond. CleaveNet Generated	0.98	0.11	0.20	0.43	0.37	24/24
mRNA-disp. efficient	0.46	0.12	0.14	0.24	0.22	4/4
mRNA-disp. negative	0.00	0.00	0.00	0.00	0.00	0/4
Cond. Site-independent	0.08	0.00	0.03	0.01	0.00	2/8
Cond. Site-independent + CleaveNet Pred.	0.35	0.05	0.09	0.19	0.18	8/8
Cond. CleaveNet Generated	0.57	0.04	0.15	0.22	0.21	24/24
mRNA-disp. selective	0.16	0.05	0.05	0.10	0.09	4/4

Supplementary Table 4: **Summary of novelty features for top MMP13-efficient substrates identified.** Sequences for each substrate are provided, with the longest shared k-mer highlighted in blue. Substrates are ranked by their *in vitro* efficiencies (Eff.). The maximum Levenshtein similarity (Max. Similarity), length of the longest shared k-mer (Shared k-mer length), and maximum Z-score attained for MMP13 by a substrate sharing the longest k-mer (Max $\hat{Z}_{13}$ Training) are reported. The range of cleavable Z-scores in the mRNA display set was 1-3.32.

Substrate	Sequence	Eff.	Max. Similarity	Shared k-mer length	Max $\hat{Z}_{13}$ training
DL73	LFPLAMMDMT	1.00	70	4	2.57
DL6	IAPLGLTASG	0.98	80	8	2.29
DL50	LAPLGLTTSK	0.82	60	5	2.29
DL5	TAPLGIRDGT	0.81	70	6	1.99
DL52	VRPLAMYDLV	0.79	70	4	2.57
DL49	VAPLPMRAWL	0.73	70	5	0.50
DL3	LGPLGITASG	0.62	80	7	1.05

Supplementary Table 5: **Summary of sequence novelty for substrates generated in silico for MMP9 selectivity**. Closest sequence match in the training set, determined by Levenshtein distance, to the top MMP9 selective substrates, conditionally designed by CleaveNet.

Conditionally generated sequence	Closest training match	Levenshtein distance
VVVIAVLQIM	VTVIALLRGQ	5
VQFVAVMSTV	VFVRALISTG	5
PLPFAGVAFM	PFWFALVAKG	5
FLPLGVGVAL	FYPRNIGVAL	4
QGPFVVVGLA	SGPQAVVKTA	5
VMVRVTMVMV	VMVRFLMNQQ	5
MFVMGVFATV	PRVMLLFATG	5
LVVPAVVVMI	LVVAALVNVI	4
AGVVMLVGIM	SGPVMLRGTA	5
FPKMGVMGLV	PKMSHLRGGV	6

Supplementary Table 6: **$\hat{Z}$-score cleavage thresholds predicted with the CleaveNet transformer predictor and LSTM and respective cleavage ROC-AUCs for the 95 substrates screened *in vitro***. Threshold values for either model should be utilized when trying to assess whether a substrate is cleaved or not, given a predicted $\hat{Z}$-score.

Protease	Threshold Transformer	Threshold LSTM	ROC-AUC Transformer
MMP1	2.4	2.1	0.94
MMP2	1.0	1.5	0.86
MMP3	1.0	1.5	0.90
MMP7	0.9	1.0	0.95
MMP8	1.3	1.0	0.93
MMP9	0.3	0.5	0.77
MMP10	0.8	0.9	0.94
MMP12	0.4	0.1	0.81
MMP13	1.0	1.0	0.98
MMP14	1.0	1.3	0.89
MMP17	1.0	1.1	0.95
MMP20	0.7	1.2	0.94

Supplementary Table 7: **Summary statistics of *in vitro* MMP13 selectivity metrics by group.** "Uncond." and "Cond." represent unconditionally and conditionally, respectively.

Group	Max.	Min.	StdDev	Average	Median
Uncond. Site-independent	0.86	-0.74	0.46	-0.09	-0.14
Uncond. Site-independent + CleaveNet Pred.	1.58	-0.82	0.81	0.66	0.91
Uncond. CleaveNet Generated	2.69	-1.24	0.78	1.06	1.02
mRNA-disp. efficient	1.78	-0.63	0.94	0.34	-0.07
mRNA-disp. negative	0.00	0.00	0.00	0.00	0.00
Cond. Site-independent	3.18	-0.29	1.13	0.40	0.00
Cond. Site-independent + CleaveNet Pred.	3.05	0.34	1.10	1.76	2.14
Cond. CleaveNet Generated	3.18	0.62	0.88	2.34	2.66
mRNA-disp. selective	3.18	1.71	0.74	2.56	2.94

Supplementary Table 8: **Summary of novelty features for high efficiency-high selectivity MMP13 substrates.** Sequences for each are provided, with the longest shared k-mer highlighted in blue. Substrates are ranked by their *in vitro* MMP13 selectivities (Sel.). We report the *in vitro* MMP13 efficiency, maximum Levenshtein similarity (Max. Similarity), the length of the longest shared k-mer, the maximum MMP13 $\hat{Z}$-score (range 1-3.32) and maximum MMP13 selectivity score (range -2.15-2.74) attained by a substrate sharing the longest k-mer in training are reported.

Substrate	Sequence	Eff.	Sel.	Max. Similarity	Shared k-mer length	Max Z_{13} training	Max S_{13} training
DL48	FAPRQMFDPG	0.57	3.09	60	4	1.41	0.96
DL45	VAPYGFRTAS	0.56	2.74	70	5	1.76	1.34
DL16	PVPLGVRDTA	0.51	2.69	70	4	1.88	2.26
DL42	TIPYGFWGPY	0.46	2.52	60	4	1.96	1.72
DL5	TAPLGIRDGT	0.81	2.46	70	6	1.99	1.35

Supplementary Table 9: **Classification performance comparison between ProsperousPlus and CleaveNet.** Both tools were utilized to classify (cleaved vs. non-cleaved) the 95 substrates used in the *in vitro* fluorogenic screen against the 8 human MMPs available in ProsperousPlus. While ProsperousPlus achieved comparable sensitivity for cleavage to CleaveNet (average sensitivity of 91.3+/-15.5% vs. 90.7+/-4.49% for ProsperousPlus and CleaveNet, respectively), it demonstrated substantially lower specificity values (22.2+/-13.2% vs. 88.2+/-14.6% for ProsperousPlus and CleaveNet, respectively) and thus resulted in lower accuracy than CleaveNet (51.9+/-9.1% vs. 90.7+/-5.1% for ProsperousPlus and CleaveNet, respectively).

	ProsperousPlus	CleaveNet	ProsperousPlus	CleaveNet	ProsperousPlus	CleaveNet
**Protease**	**Sensitivity**		**Specificity**		**Accuracy**	
**MMP1**	100	88.5	20.3	98.6	42.1	95.8
**MMP8**	58.5	87.8	50	100	53.7	94.7
**MMP9**	100	95.2	12.1	60.6	69.5	83.2
**MMP7**	84.4	93.9	23.8	96.8	44.2	95.8
**MMP2**	100	95.5	15.7	76.5	54.7	85.3
**MMP3**	100	83.7	11.5	96.2	51.6	90.5
**MMP14**	96.9	90.6	22.2	88.9	47.4	89.5
**Average**	91.3	90.7	22.2	88.2	51.9	90.7
