# Supplementary material for: Deep learning guided design of protease substrates
Source: Nat Commun. 2026 Jan 6;17:54. doi: 10.1038/s41467-025-67226-1 (PMC12775482; doi:10.1038/s41467-025-67226-1)
Supplement: Supplementary file 2 — Description of Additional Supplementary Files [file 41467_2025_67226_MOESM2_ESM.pdf]

Description of Additional Supplementary Files

File Name: Supplementary Data 1
Description: Full list of sequences for FRET-paired substrates used in the in vitro screen. Sequences include N- and C-terminal modifications.
