# Supplementary material for: Deep learning guided design of protease substrates
Source: Nat Commun. 2026 Jan 6;17:54. doi: 10.1038/s41467-025-67226-1 (PMC12775482; doi:10.1038/s41467-025-67226-1)
Supplement: Supplementary file 4 — Reporting Summary [file 41467_2025_67226_MOESM4_ESM.pdf]

nature portfolio

Corresponding author(s): Ava P. Amini

Last updated by author(s): Nov 17, 2025

Reporting Summary

Nature Portfolio wishes to improve the reproducibility of the work that we publish. This form provides structure for consistency and transparency in reporting. For further information on Nature Portfolio policies, see our Editorial Policies and the Editorial Policy Checklist.

Statistics

For all statistical analyses, confirm that the following items are present in the figure legend, table legend, main text, or Methods section.

n/a	Confirmed	
☐	☒	The exact sample size (*n*) for each experimental group/condition, given as a discrete number and unit of measurement
☒	☐	A statement on whether measurements were taken from distinct samples or whether the same sample was measured repeatedly
☐	☒	The statistical test(s) used AND whether they are one- or two-sided<br>*Only common tests should be described solely by name; describe more complex techniques in the Methods section.*
☒	☐	A description of all covariates tested
☐	☒	A description of any assumptions or corrections, such as tests of normality and adjustment for multiple comparisons
☐	☒	A full description of the statistical parameters including central tendency (e.g. means) or other basic estimates (e.g. regression coefficient) AND variation (e.g. standard deviation) or associated estimates of uncertainty (e.g. confidence intervals)
☐	☒	For null hypothesis testing, the test statistic (e.g. *F*, *t*, *r*) with confidence intervals, effect sizes, degrees of freedom and *P* value noted<br>*Give P values as exact values whenever suitable.*
☒	☐	For Bayesian analysis, information on the choice of priors and Markov chain Monte Carlo settings
☒	☐	For hierarchical and complex designs, identification of the appropriate level for tests and full reporting of outcomes
☐	☒	Estimates of effect sizes (e.g. Cohen's *d*, Pearson's *r*), indicating how they were calculated

Our web collection on statistics for biologists contains articles on many of the points above.

Software and code

Policy information about availability of computer code

Data collection	No software was used for data collection.
Data analysis	All code, model weights, generated sequences, and computed metrics are available at https://github.com/microsoft/cleavenet. Custom code for analysis is described in the manuscript Methods.

For manuscripts utilizing custom algorithms or software that are central to the research but not yet described in published literature, software must be made available to editors and reviewers. We strongly encourage code deposition in a community repository (e.g. GitHub). See the Nature Portfolio guidelines for submitting code & software for further information.

Data

Policy information about availability of data

All manuscripts must include a data availability statement. This statement should provide the following information, where applicable:

- Accession codes, unique identifiers, or web links for publicly available datasets
- A description of any restrictions on data availability
- For clinical datasets or third party data, please ensure that the statement adheres to our policy

The raw data for this study and the data generated in this study have been deposited in a GitHub repository and are freely available at https://github.com/microsoft/cleavenet. The source data for individual figures are provided in the Source Data file.

Research involving human participants, their data, or biological material

Policy information about studies with human participants or human data. See also policy information about sex, gender (identity/presentation), and sexual orientation and race, ethnicity and racism.

Reporting on sex and gender	N/A
Reporting on race, ethnicity, or other socially relevant groupings	N/A
Population characteristics	N/A
Recruitment	N/A
Ethics oversight	N/A

Note that full information on the approval of the study protocol must also be provided in the manuscript.

Field-specific reporting

Please select the one below that is the best fit for your research. If you are not sure, read the appropriate sections before making your selection.

[x] Life sciences [ ] Behavioural & social sciences [ ] Ecological, evolutionary & environmental sciences

For a reference copy of the document with all sections, see nature.com/documents/nr-reporting-summary-flat.pdf

Life sciences study design

All studies must disclose on these points even when the disclosure is negative.

Sample size	Sample sizes were chosen to power in silico and in vitro analyses. For in silico analyses, sample sizes were matched to facilitate comparison between methods. For in vitro analyses, sample sizes were also matched between design conditions to facilitate powered comparison between methods.<br><br>Replicates of fluorogenic assays between FRET substrates and recombinant proteases demonstrate relatively low standard deviation. Additionally, data is collected longitudinally with cleavage leading to monotonic increases over time, further increasing the confidence in the data. For these reasons, our laboratory has deemed technical duplicates to be appropriate to evaluate cleavage across individual protease-substrate pairs (Kirkpatrick et al., 2020, Zhong et al., 2024). Two independent runs with different peptide plates and protease preparation (new commercial vial, new buffer) were performed to ensure reproducibility of the data.
Data exclusions	No data was excluded.
Replication	Computational models were trained across 5 independent random seeds and 5 independent train-validation splits, and evaluated using the independent test sets, to ensure reproducibility and facilitate ensembling for greater robustness.<br><br>In vitro experiments were repeated independently for reproducibility (total of 2 independent executions). Two technical replicates were additionally included in the in vitro screens. All attempts at replication were successful.
Randomization	Peptide sequences were allocated into experimental groups based on how they were designed or selected (i.e., CleaveNet-generated, positive controls, negative controls, etc). Procedure for sequence design and/or selection are detailed in the manuscript Methods. Sample randomization was not relevant to this study.
Blinding	Investigators were not blinded to experimental conditions; experimental setup and execution necessitated knowledge of the experimental conditions.

Reporting for specific materials, systems and methods

We require information from authors about some types of materials, experimental systems and methods used in many studies. Here, indicate whether each material, system or method listed is relevant to your study. If you are not sure if a list item applies to your research, read the appropriate section before selecting a response.

Materials & experimental systems

n/a	Involved in the study
☒	☐ Antibodies
☒	☐ Eukaryotic cell lines
☒	☐ Palaeontology and archaeology
☒	☐ Animals and other organisms
☒	☐ Clinical data
☒	☐ Dual use research of concern
☒	☐ Plants

Methods

n/a	Involved in the study
☒	☐ ChIP-seq
☒	☐ Flow cytometry
☒	☐ MRI-based neuroimaging

Plants

Seed stocks	N/A
Novel plant genotypes	N/A
Authentication	N/A
